# SIBLINGS AFFECTED BY ECTRODACTYLY-ECTODERMAL DYSPLASIA AND CLEFT LIP/PALATE (EEC) SYNDROME PRESENTING NORMAL PARENTS: GERMLINE MOSAICISM?

**DOI:** 10.1590/1984-0462/;2017;35;2;00017

**Published:** 2017-05-15

**Authors:** Rafael Fabiano Machado Rosa, Samir Abou Ghaouche de Moraes, Leonardo Paludo Sulczinski, Filipe Augusto da Silva, Olga Gaio Milner, Silvana Rodrigues Streit Pires, Osvaldo Alfonso Pinto Artigalas, Rosana Cardoso Manique Rosa, Paulo Ricardo Gazzola Zen

**Affiliations:** a Universidade Federal de Ciências da Saúde de Porto Alegre (UFCSPA), Porto Alegre, RS, Brasil.; b Hospital Materno-Infantil Presidente Vargas (HMIPV), Porto Alegre, RS, Brasil.

**Keywords:** Cleft lip, Extremities, Genetics, Heredity, Mosaicism

## Abstract

**Objective:**

EEC is an acronym for an autosomal dominant syndrome clinically characterized by ectrodactyly (E), ectodermal dysplasia (E) and cleft lip/palate (C). Our aim was to describe a rare case of siblings affected by ectrodactyly, ectodermal dysplasia and cleft lip/palate (EEC) syndrome presenting normal parents.

**Case description:**

The patient was the third son of young and healthy parents. The parents did not present any minor or major anomaly of hands, feet or skin, hair and teeth. The couple had a previous history of two children with hands and feet malformations similar to the present patient. The first was a stillborn, and the second one a preterm infant that died in the first days after birth due to the consequences of prematurity. After birth, the patient presented respiratory distress with need of endotracheal intubation and mechanic ventilation. At physical examination, there were cleft lip/palate, hands and feet ectrodactyly, with absence of the second and third fingers in both hands, and reduction defects affecting mainly the second toes. The child presented pneumothorax and cardiorespiratory arrest and died at 1 month and 26 days.

**Comments:**

Herein we described a case of siblings with EEC syndrome, indicative of a germline mosaicism. In the literature review, there was the description of only three similar reports. The present case strengthens the possibility that germline mosaicism may be a more common inheritance mechanism than previously thought in cases of EEC syndrome.

## RESUMO

**Objetivo:**

EEC é um acrônimo para uma síndrome autossômica dominante caracterizada clinicamente por ectrodactilia (E), displasia ectodérmica efissura labiopalatal (C). Nosso objetivo foi relatar um caso raro de irmãos afetados pela síndrome de ectrodactilia, displasia ectodérmica efissura labiopalatal (EEC) com pais hígidos.

**Descrição do caso:**

O paciente era o terceiro filho de pais jovens e hígidos, os quais não apresentavam nenhuma anomalia menor ou maior de mãos e pés ou anomalias de pele, cabelos e dentes. O casal tinha história prévia de duas crianças com malformação de mãos e pés, similar à do paciente. O primeiro foi natimorto e o segundo, prematuro, falecendo nos primeiros dias de vida, pelas consequências da prematuridade. Após o nascimento, o paciente apresentou desconforto respiratório, com necessidade de intubação orotraqueal e ventilação mecânica. No exame físico, verificaram-se a presença de fissura labiopalatal e ectrodactilia de mãos e pés, com ausência do segundo e terceiro dedos em ambas as mãos e defeitos de redução acometendo principalmente o segundo dedo dos pés. A criança apresentou pneumotórax e parada cardiorrespiratória, morrendo com 1 mês e 26 dias de vida.

**Comentários:**

Descrevemos aqui um caso de irmãos com síndrome EEC, indicativo de mosaicismo germinativo. Na revisão da literatura, observaram-se apenas três relatos similares. Este caso reforça a possibilidade do mosaicismo germinativo ser um mecanismo de herança mais comum do que se acreditava previamente para casos da síndrome EEC.

## INTRODUCTION

Ectrodactyly, also known as lobster-claw defect or split hand-foot, is a form of congenital absence of one or more digits that involves the central rays of the hand and foot. EEC is an acronym for a syndrome characterized by ectrodactyly (E), ectodermal dysplasia (E) and cleft lip/palate (C). This syndrome has an autosomal dominant pattern of inheritance with a high penetrance and is part of the group of syndromes called “split hand-foot malformations” (split hand/split foot malformation, SHFM, - Online Mendelian Inheritance in Man, OMIM, 183600), which encompasses conditions characterized by distal abnormalities of limbs with different degrees of severity.^1^ Two types of EEC, EEC1 (OMIM 129900) and EEC3 (OMIM 604292), have been described with mutations in genes localized on chromosome 7q11 and 3q28 (that encodes the *p63* gene), respectively.

The main clinical findings of EEC are varying degrees of mesoaxial and longitudinal defects in the distal part of the limbs, cleft lip and palate, and defective development of ectodermal derivatives which appear as malformed or missing teeth, dystrophic nails, lacrimal duct stenosis, thin nipples and underdeveloped, absent or hypopigmented hair. Additional manifestations include hearing impairment and urinary tract anomalies.^1^

Our aim was to describe a rare case of siblings affected by EEC syndrome presenting normal parents.

## CASE DESCRIPTION

**Figure 1:**
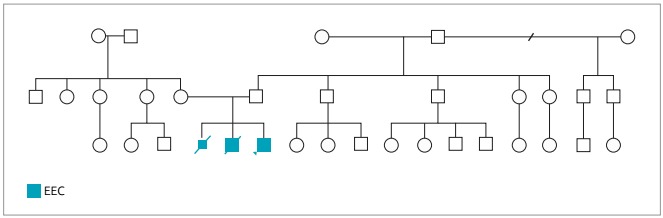
Pedigree of the family showing the individuals affected by EEC syndrome, indicative of germline mosaicism.

The patient was the third son of young and healthy parents.The parents did not present any minor or major anomaly of hands and feet, or even skin, hair and teeth abnormalities. The couple had a previous history of two children with hands and feet malformations (compatible with ectrodactyly) similar to the patient. The first one was a stillborn, and the second one a preterm infant, that died in the first days after birth due to consequences of prematurity. Family history was negative for other individuals with similar malformations (Figure 1). The mother denied threats of abortion, usage of illegal medications or drugs, and alcohol intake during pregnancy. She smoked six cigarettes a day.

**Figure 2:**
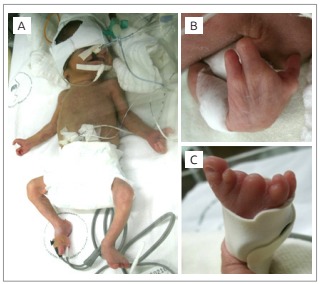
Clinical features presented by the patient. Note especially cleft lip (A) and hands (A and B) and feet (A and C) ectrodactyly.

The patient was born by cesarean section, at 32 weeks of gestation, weighing 1,140g and with an Apgar score of 2 at first minute and 4 at fifth minute. After birth, the child presented respiratory distress with need of endotracheal intubation and mechanic ventilation. At physical examination, it was verified the presence of: bilateral cleft lip and complete cleft palate (involving hard and soft palate), micrognathia, abnormal low-set ears, short neck, and limb defects affecting the central ray of the hands and feet, compatible with ectrodactyly. The second and third fingers in both hands were absent, and there were reduction defects affecting mainly the second toes. Hands and feet radiographies revealed absence of phalanges of the second and third fingers, alteration of the second metacarpal bone of the left hand and of the phalanges of the first finger. A morphologic alteration of the second toe in the left foot and of toes in the right foot was also noted (Figure 2 and Table 1).

**Table 1:**
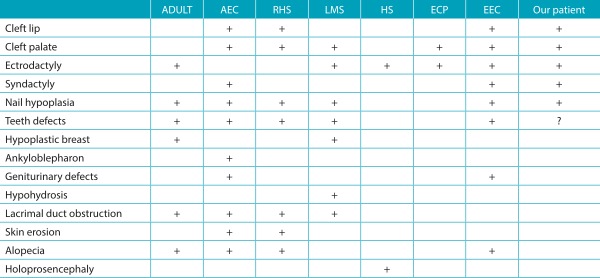
Comparative findings between our case and *p63* associated conditions and split hand-foot malformations (SHFM) type syndromes.

ADULT: acro-dermato-ungual-lacrimal-tooth syndrome; AEC: ankyloblepharon-ectodermal dysplasia-clefting; RHS: Rapp-Hodgkin syndrome; LMS: limb mammary syndrome; HS: Hartsfield syndrome; ECP: ectrodactyly-cleft palate syndrome; EEC: ectrodactyly, ectodermal dysplasia and cleft lip/palate syndrome; ?: unknown.

Abdominal ultrasound and high resolution G bands produced with Trypsin and Giemsa (GTG)-Banding karyotype were normal. Echocardiography revealed a patent foramen ovale. Brain ultrasound showed dilated ventricles, paraventricular cysts and multiple frontoparietal cystic areas, suggestive of leukomalacia due to a hypoxic ischemic event. Ophthalmological examination disclosed signals of retinopathy of prematurity in the right eye (Table 1).

The patient presented pneumothorax and cardiorespiratory arrest in the first week of life and died at 1 month and 26 days.

## DISCUSSION

The findings of our patient, with the presence of hands and feet ectrodactyly associated to cleft lip and palate, were compatible with the diagnosis of EEC syndrome (Table 1). EEC is the most frequent syndromic form of SHFM, but other entities have been described, some of which are allelic to EEC, reflecting the pleiotropic effects of mutations in the *p63* gene. Heterozygous mutations in the transcription factor gene *p63* are causative of several syndromes. These syndromes have ectodermal dysplasia, orofacial clefts and limb malformations as key characteristics, because this gene is an important regulator of orofacial, ectodermal and limb development,^2^ and include: limb mammary syndrome (LMS) (OMIM 603543), acro-dermato-ungual-lacrimal-tooth syndrome (ADULT) (OMIM 103285), Hay-Wells syndrome/ankyloblepharon-ectodermal dysplasia-clefting (AEC) (OMIM 106260), and Rapp-Hodgkin syndrome (RHS) (OMIM 129400)^1^ (Table 1).

The clinical findings of our patient are not consistent with LMS due to absence of breast and/or nipple hypoplasia, a key feature in this syndrome, verified in all reported patients.^2^ Additionally, lacrimal duct obstruction and hypohidrosis are also frequent in LMS and absent in our case. Similarly, our patient did not present ADULT syndrome because orofacial clefts, a feature present in our case, do not belong to the spectrum of this condition.^2^ ^,^ ^3^ The clinical presentation of AEC syndrome is marked by fusion of upper and lower eyelids at birth due to bands of fibrous tissue and absence of ectrodactyly. Lacrimal duct obstruction and genitourinary defects are also common findings,^2^ ^,^ ^3^ and these features were not present in our patient. RHS also differs from the reported case due to the absence of limbs malformations (as ectrodactyly).^2^ ^,^ ^3^

SHFM can also be found as an isolated feature or associated to other syndromes that are not related to *p63*, such as ectodermal dysplasia, ectrodactilia and macular dystrophy (EEM) (OMIM 225280) and acro-cardio-facial syndrome (ACFS) (OMIM 600460). However, both conditions show an autosomal recessive pattern of inheritance^4^ ^,^ ^5^ (Table 1). Furthermore, EEM is distinguished from other SHFM syndromes by the presence of some characteristic ocular findings, such as retinochoroidal atrophy, with extensive pigmentation of the retina and arteriolar attenuation on the posterior pole. These findings were absent in our case, that had only a retinopathy associated to the prematurity. ACFS is characterized by SHFM associated with complex cardiac malformations and genitourinary defects, which were not verified in our patient. Thus, despite the fact that the family pedigree of the patient reported here may suggest a recessive pattern of inheritance, he did not present clinical findings compatible with ACFS or EEM.

Other conditions associated to ectrodactyly to be considered in the differential diagnosis of our patient include Hartsfield syndrome (OMIM 615465) and ectrodactyly-cleft palate (ECP) syndrome (OMIM 129830). Hartsfield syndrome is a rare genetic condition associated to mutations in the *FGFR1* gene. It is characterized by the presence of ectrodactyly and cleft lip and palate. However, holoprosencephaly, a feature not seen in our patient, is considered a fundamental finding.^6^ Ectrodactyly-cleft palate syndrome is a condition described based on a single large family presenting ectrodactyly and cleft palate, without other clinical manifestations described in EEC syndrome.^7^ It is noteworthy that, despite the description of oral clefts, these are limited to the palate in ECP syndrome,^7^ different from our patient, that had involvement of the lip (Table 1).

Giannotti et al.^8^ also reported a boy with some similar characteristics, including bilateral ectrodactyly. He was born of healthy and non-consanguineous parents who had a history of a previous gestation with the preterm birth of a girl presenting similar findings. However, the patient did not present ectodermal involvement and had congenital heart defects and genital abnormalities (hypospadia, micropenis and hypoplastic scrotum, without palpable testes) which differentiate him from EEC syndrome. The case reported by Gianotti et al.^8^ was fairly similar to one described in Brazil by Richieri-Costa et al.^9^ The authors suggested that their findings revealed a possible autosomal recessive syndrome.^8^

In this context, despite the fact that some features of ectodermal dysplasia (as teeth abnormalities) could not be properly evaluated due to the early age, our patient probably had EEC syndrome. His diagnosis was based especially on identification of limb abnormalities affecting the central rays of hands and feet, giving rise to the lobster or ectrodactyly, and on the presence of oral clefts affecting not only the palate, but also the lip. It is noteworthy, in the family of the reported patient, the presence of siblings affected by EEC syndrome and normal parents, without other cases in the rest of the family. 

Despite the highly variable expressivity reported in EEC syndrome, it is a known condition to have high penetrance.^2^ Thus, it would be extremely rare for a new mutation to occur in all children of the reported family. The most likely cause for three affected siblings is that one of the parents presents a germline mosaicism for EEC syndrome. *Mosaic* is a term that has been used to refer to an artistic patchwork composed, for example, by ornamental stones, glass, or gems.^10^ Germline mosaicism, also known as gonadal mosaicism, is used to indicate the presence of genetically distinct populations, due to DNA mutations, epigenetic alterations of DNA, or chromosomal abnormalities, in germ-line/gonadal tissues.^10^ ^,^ ^11^ However, germline mosaicism is difficult to detect, because germ-line DNA is difficult to access, especially in females.^10^ ^,^ ^12^ Therefore, the germline mosaicism diagnosis usually tends to be based primarily on the clinical information that is obtained from examining both the proband and the offspring.^10^ Germline mosaicism has been described involving genetic abnormalities associated to gene diseases with different patterns of inheritance. It has been also thought to be a rare cause of chromosomal anomalies, as aneuploidies (for example, trisomy 21), in human population.^12^ Germline mosaicism is most commonly described with X-linked and autosomal dominant disorders, as EEC syndrome. Even autosomal recessive disorders, as Alport syndrome, may be involved.^13^ Hartl^14^ suggests that, if one of the parents presents a presumptive germinal mosaicism and has more than one affected child, the risk to subsequent offspring varies from 20 to 35%. This value would vary depending on the number of normal children among the siblings. These findings strengthen the hypothesis of the presence of germline mosaicism in the family reported here.

In the literature review, there was the description of three reports of germline mosaicism in families with EEC syndrome. David^15^ reported individuals born of unaffected and non-consanguineous parents who presented ectrodactyly affecting all limbs. These individuals had at least two affected children who also presented affected descendants in all following described generations. Barrow et al.^16^ described a case in which apparently normal parents originate an affected lineage. Their two children had ectodermal dysplasia, with sparse to absent blonde hair, eyebrows, and eyelashes, dysplastic nails, palmar hyperkeratosis, and cone shaped teeth. They also had renal abnormalities and one of them presented a cleft palate. One individual had an affected son presenting similar features. A splice somatic mutation in *p63* was detected in all affected individuals, but not in their parents.^16^ Kosaki et al.^17^ reported a boy with EEC syndrome whose father had somatic and germline mosaicism for mutations in *p63* gene. He presented partial gray hair, enamel hypoplasia with partial anodontia, ectrodactyly of hands, with some trapezoidal shaped fingernails, and mildly hyperpigmented patches following the Blashko lines in upper extremities.^17^

Thus, we have reported a case of siblings with EEC syndrome, suggestive of germline mosaicism. Our case strengthens the possibility that the germline mosaicism may be a more common inheritance mechanism than previously thought in cases of EEC syndrome.
